# Partly unequal receipt of healthcare in last month of life in amyotrophic lateral sclerosis: a retrospective cohort study of the Stockholm region

**DOI:** 10.48101/ujms.v129.9856

**Published:** 2024-02-07

**Authors:** Peter Strang, Torbjörn Schultz, Anneli Ozanne

**Affiliations:** a Department of Oncology-Pathology, Karolinska Institutet, Stockholm, Sweden; b Research and Development Department, Stockholm’s Sjukhem Foundation, Stockholm, Sweden; c Institute of Health and Care Sciences, Sahlgrenska Academy, University of Gothenburg, Gothenburg, Sweden; d Department of Neurology, Sahlgrenska University Hospital, Gothenburg Sweden

**Keywords:** Amyotrophic lateral sclerosis, emergency room visits, healthcare utilization, palliative care, place of death, terminal care

## Abstract

**Context:**

In amyotrophic lateral sclerosis (ALS), equal care is important, given that the disease often has complex symptoms at the end of life.

**Objectives:**

The aim was to study the possible associations between demographic and clinical factors, including age, sex, and frailty, with acute healthcare utilization in the last month of life, measured by emergency room (ER) visits, admissions to acute hospitals and, acute hospitals as place of death, among patients with ALS. A second aim was to study whether receipt of specialized palliative care (SPC) affects above-mentioned healthcare utilization.

**Methods:**

Observational, retrospective study based on Region Stockholm’s administrative data warehouse (VAL) in Sweden. Data were retrieved for 2015–2021 and analyzed with descriptive statistics and logistic regression models.

**Results:**

All deceased patients (*n* = 448) ≥18 years with ALS were included. The mean age was 70.5 years, 46% were women and 58% had risk of frailty according to Hospital Frailty Risk Score (HFRS). Ninety-nine (22%) were nursing home residents and 49% received SPC. The receipt of SPC in patients with ALS was equal in relation to gender, socio-economic standing, frailty, and age <75 years. Patients ≥75 years, those with dementia and/or residing in nursing homes (NH) were less likely to receive SPC (*P* = 0.01, *P* = 0.03 and *P* = 0.002, respectively). Receipt of SPC reduced ER visits (29% vs. 48%, *P* < 0.001) and deaths at hospital (12% vs. 48%, *P* <0.001). Patients who were frail, had a higher risk of ER visits and were more likely to die at an acute hospital setting (*P* < 0.001 and *P* = 0.004). NH residents were less likely to have ER visits and to die in hospital (*P* = 0.002 and *P* = 0.005).

**Conclusions:**

The results indicate partly unequal distribution of palliative care, however the actual, individual preferences cannot be deducted from registry studies. All patients with ALS should be offered SPC when needed.

**Key message:**

This register study shows that receipt of SPC in patients with ALS is equal in relation to gender, socioeconomic standing, frailty, and age <75 years, while those ≥75 years, with dementia, or residing in NH were somewhat less likely to receive SPC. Receipt of SPC reduces ER visits and acute hospital admissions.

## Introduction

Amyotrophic lateral sclerosis (ALS) is a degenerative, motor neuron disease (MND), with an average survival of 2–5 years from the first symptoms. Muscle atrophy, weakness and spasticity, with paralysis of the extremities, the swallowing-, speech-, and breathing muscles are common and affect the whole life situation (1). Around 35–45% also suffer from cognitive impairment or dementia (10–15%) (2).

Today, no cure exists, focus is on prolonging the survival, on symptom relief, and on optimizing the quality of life (3, 4). This means that the focus of care can be considered palliative already at diagnosis. Symptoms gradually increase with deterioration of function and nutritional status, but in different rates and degrees of severity (5). At the end of life, symptoms such as dyspnea, rattles, anxiety, restless legs, and pain are often described as problematic (5–8). The life situation creates emotional and existential distress, and affects quality of life, in both patients and relatives (9–13). The trajectory towards death might be difficult with insufficient symptom relief, therefore, access to specialized palliative care (SPC) can be valuable (14, 15). Death often occurs peacefully (7, 16) and around 6% have a sudden death, often dependent on heart-related causes (17). In medical records, the most frequently reported cause of death is respiratory failure/hypoxic death (7), and in the autopsy, broncho-pneumonia is the predominant diagnosis (18).

Place of death varies, as patients may die in their own home with or without specialist palliative care or at a palliative unit (5, 7, 8, 16). However, a French study by Gil et al. showed that 63% died in a medical facility (mostly in a department of neurology, emergency unit, department of pulmonary medicine, functional rehabilitation, and long-term care facility or palliative care unit) and 37% outside a medical facility (of which 98% died at home) (19). A study from USA found that 50% died at home or in a hospice facility, 25% died in an acute care facility, and 20% died at a nursing home. Furthermore, minorities, male and unmarried patients more often died in an acute care facility than other patients with ALS (20). In Sweden, national palliative quality register studies show that 30–34% die at hospital, around 35% die at home with the aid of specialized palliative home care or at an inpatient palliative care unit (6, 8), and about 21% die in nursing homes (6).

The multiprofessional team is an important part of the care during the course of the disease and has a positive effect on the patient satisfaction and outcome (1). However, the constellation of the team and possible collaboration with specialized palliative care (SPC) differ. In Sweden, most patients with ALS are treated and followed up by an ALS/motor neuron disease (MND)-team, although the constellation of the team members and accessibility differ. Later in the trajectory, SPC is provided in the form of advanced palliative home care, and/ or by hospital palliative care units. The SPC teams are multiprofessional, with physicians, registered nurses, physiotherapists, occupational therapists, dieticians, and assistant nurses (21). When needed, people enrolled in SPC have access to medical aids such as cough machines to facilitate expectorations, speech synthesis devices, non-invasive ventilation (NIV), tube feeding and so on and SPC teams are specialists on symptom control.

In order to provide equal care, it is not only necessary to explore how and where the care is conducted during the last year of life but also during the critical last month of life, whether socioeconomic factors affect receipt of care, and whether SPC reduces the likelihood of emergency hospitalization. The aim was to study possible associations between demographic and clinical factors, including age, sex, frailty, and acute healthcare utilization in the last month of life, measured by emergency room (ER) visits, admissions to acute hospitals and, acute hospitals as place of death, among patients with ALS. A secondary aim was to study whether receipt of SPC correlates with above-mentioned healthcare utilization.

## Patients and methods

Methods and Results are reported based on the Strengthening the Reporting of Observational Studies in Epidemiology (STROBE) criteria (22).

### Study design and setting

This observational retrospective study was based on Region Stockholm’s administrative data warehouse (VAL) in Sweden and data were retrieved for 2015–2021. The registry comprises caregiver-provided data from all hospital visits, appointments, in-ward care episodes, diagnoses according to WHO ICD-10 classification. Reporting to the VAL database is mandatory to all caregivers funded by the Stockholm region, thus offering a complete data on all healthcare being offered, with very few missing values. The Swedish healthcare system is funded by taxes and publicly available to all its citizens.

### Population

All deceased patients 18 years and older with a diagnosis of ALS (motor neuron disease G12.2 according to ICD-10 classification) were included.

### Variables

We used ER visits, and hospital deaths as outcome measures. As explanatory variables we used age, sex, frailty as measured by the Hospital Frailty Risk Score (HFRS) (23), socio-economic Mosaic groups (24, 25), dementia, receipt of SPC or being a nursing home (NH) resident.

HFRS is a validated measure of frailty, based on 109 weighted ICD-10 diagnoses, diagnoses that have been found to be more prevalent in frail persons (23). The lookback window was 1 year from the time of death for each of the included patients.

Mosaic is a commercial socio-economic measure on an area level, to which Stockholm Region subscribe (24, 25). With the aid of Mosaic, Stockholm County is divided into 1,200–1,300 small areas and labelled as Mosaic 1, 2 or 3, not only on the basis of socio-economic variables such as income and education but also on more than 40 additional variables including living arrangements, cultural aspects and lifestyle. Mosaic group 1 areas are the most affluent ones, whereas Mosaic group 3 comprises less affluent areas. In this analysis, we merged Mosaic group 1+2, which we compared with Mosaic group 3.

Diagnoses of dementia were identified by using the ICD-10 codes F00-F03.

### Bias

Dropouts: Since the inclusion is based on ICD-10 codes and data is mandated to report and the base of the caregivers’ economic compensation, missing data are very few (missing values are estimated to be <1%).

### Study size

As a total cohort (all patients with ALS who died between 2015 and 2021) was studied, no power calculations were made.

### Statistical methods

T-tests were used for comparison of means and substituted with Wilcoxon Rank Sum test (Mann–Whitney U test) for comparisons with skew distributions, Chi-square tests were applied for comparison of proportions. Initially, univariable logistic regression analyses were performed for relevant variables, which then were entered into fully adjusted logistic regression models and adjusted Odds ratios (aORs) were calculated. As a measure of goodness of fit for binary outcomes in our multiple logistic regression models, we calculated C-statistic (equivalent to the area under the curve [AUC], in this case the area under the receiver operatring characteristic [ROC] curve). A C statistic value of 0.5 indicates that the model is no better than chance at making a prediction of membership in a group and a value of 1.0 indicates that the model perfectly identifies those within a group and those not. The SAS 9.4/Enterprise guide 8.2 was used for carrying out data analyses.

### Ethics

The Regional Ethical Review Authority (EPN 2017/1141‐31) approved this study.

## Results

### Demographics and clinical data

There was a total of 448 persons who died with ALS as a main diagnosis, of which 99 persons were NH residents. The mean age for the whole group was 70.5 years; 68.8 years for those in ordinary housing and significantly higher for those who were NH residents, 76.4 years (*P*<0.001) (Table 1). The gender distribution was 54% men and 46% women. A total of 58% were classified as frail according to HFRS. Forty-nine per cent received SPC. For the distribution of socio-economic status on area level, please see Table 1.

**Table 1:** Demographic and clinical data (*n* = 448, of which 99 were NH residents).

Characteristics	Total (*n* = 448)	Ordinary living (*n* = 349)	NH residents (*n* = 99)	*P*
**Age,** mean years (SD)	70.5 (10.0)	68.8 (9.8)	76.4 (8.5)	<0.001
**Age groups (years)**				<0.001
18–64, *n* (%)	115 (26)	109 (31)	6 (6)	
65–74, *n* (%)	188 (42)	146 (42)	42 (42)	
>75, *n* (%)	145 (32)	94 (27)	51 (52)	
**Sex**				0.56
Women, *n* (%)	206 (46)	163 (47)	43 (43)	
Men, *n* (%)	242 (54)	186 (53)	56 (57)	
**Mosaic** groups (SES on area level)				0.54
Group 1+2 (advantaged groups), *n* (%)	310 (61)	244 (70)	66 (67)	
Group 3 (less advantaged groups), *n* (%)	138 (31)	105 (30)	33 (33)	
**HFRS (frailty) score** ^1^				0.006
HFRS, low risk (group 1), *n* (%)	190 (42)	160 (46)	30 (30)	
HFRS, frailty risk (group 2+3), *n* (%)	258 (58)	189 (54)	69 (70)	
Dementia Specialized palliative care	32 (7)	19 (5)	13 (13)	0.009
**SPC** (Specialized palliative care last 3 months)	221 (49)	191 (55)	30 (30)	<0.001

1 The hospital frailty risk score (HFRS) divides patients into a low-risk (group 1, score <5), an intermediate risk score group (group 2, score 5–15) and a high-risk group (group 3, score >15)

### Receipt of specialized palliative care

Receipt of SPC was less likely for the oldest age-group 75 years or older, both in univariable and multivariable models. Likewise, having dementia and/ or being NH resident was associated with a lower likelihood of receiving SPC (Table 2) and in the regression model including all patients (including nursing home residents), the aOR for persons aged 75 years or more was 0.50 (0.29–0.84, *P* = 0.01), for persons with dementia 0.40 (0.18–0.93, *P* = 0.03) and for nursing home residents 0.45 (0.27–0.74, *P* = 0.002). Neither sex, socio-economic status (Mosaic), nor frailty (HRFS) were associated with receipt of SPC. For details, please see Table 2.

**Table 2:** Variables associated with receipt of SPC during the last 3 months of life among patients with ALS. Model 2a: Univariable and multivariable analyses for patients who live in their own homes (*n* = 349), NH residents were *excluded* (*n* = 99). Model 2b: Multivariable analysis for all patients, including NH residents.

Variable	Model 2a Univariable analysis. NH residents excluded, (*n* = 349)		Model 2a^e^ Multivariable analysis. NH residents excluded, (*n* = 349)		Model 2b^e^ Multivariable analysis, NH residents included, (*n* = 448)	
	OR^a^ (95% CI)	*P*	aOR^b^ (95% CI)	*P*	aOR^b^ (95% CI)	*P*
**Age groups (years)**						
18–64	Ref.		Ref.		Ref.	
65–74 >75	0.76 (0.46–1.26) 0.55 (0.32–0.96)	0.29 **0.04**	0.76 (0.45–1.27) 0.52 (0.30–0.92)	0.29 **0.03**	0.73 (0.45–1.20) 0.50 (0.29–0.84)	0.21 **0.01**
**Sex**						
Men	Ref.		Ref.		Ref.	
Women	1.25 (0.82–1.91)	0.30	1.29 (0.84–2.00)	0.24	1.37 (0.91–1.99)	0.13
**Socio-economic status**^c^ Mosaic groups 1+2 Mosaic group 3	Ref. 0.98 (0.62–1.54)	0.91	Ref. 0.96 (0.60–1.53)	0.85	Ref. 0.85 (0.56–1.30)	0.46
**HFRS**^d^ 1 (not frail) 2+3 (frail)	Ref. 0.85 (0.56–1.30)	0.46	Ref. 0.93 (0.60–1.45)	0.75	Ref. 0.95 (0.64–1.41)	0.79
**Dementia** No Yes	Ref. 0.36 (0.13–0.98)	**0.04**	Ref. 0.35 (0.13–0.98)	**0.04**	Ref. 0.40 (0.18–0.93)	**0.03**
**Nursing home resident**						
No Yes	N/A in this model		N/A in this Model		Ref. 0.45 (0.27–0.74)	**0.002**

a OR, Odds ratio;

b aOR, adjusted Odds ratio;

c Socio-economic status: Mosaic groups 1+2 are more advantaged groups, Mosaic group 3 is a less advantaged group.

d HFRS, Hospital Frailty Risk Score.

e C statistic was 0.65 and 0.60 for models 2a and 2b, respectively.

Significant values (*P*<0.05) are written in bold.

### Healthcare utilization, related to receipt of SPC

There were significant differences between those who did or did not receive SPC, as regards healthcare utilization during the last month of life. Those receiving SPC had less of unplanned ER visits, 29% versus 48% (*P*<0.001), as well as fewer acute hospital admissions, 28% versus 44% (*P*<0.001). For those who received SPC, acute hospitals were their place of death in 12% of the cases, compared to 48% for the others (*P*<0.001) (Table 3).

**Table 3:** Acute healthcare utilization during the last month of life among patients with ALS with and without receipt of specialized palliative care.

Care utilization	Total (*n* = 448)	With SPC (*n* = 221)	Without SPC (*n* = 227)	*P* ^a^
**Unplanned ER visits,** *n* (%)	174 (39)	65 (29)	109 (48)	<0.001
**Hospital admissions,** *n* (%)	163 (36)	62 (28)	101 (44)	<0.001
**Hospital as place of death,** *n* (%)	135 (30)	27 (12)	108 (48)	<0.001

a Chi-square test between those who did or did not receive SPC.

### Unplanned emergency room visits during the last month of life

In univariable and multivariable models, including age, gender, socio-economic status (Mosaic), frailty (HFRS), dementia, as well as receipt of SPC or being NH residents, only frailty, receipt of SPC or being NH resident were significantly associated with unplanned ER visits in the final, multivariable models. Frailty, as measured by HFRS, was associated with an increased likelihood of unplanned ER visits (aOR 2.22 [1.46–3.39], *P* = 0.0002). Those receiving SPC had significantly fewer unplanned ER visits in all models. As regards the model (model 4a) where only persons in ordinary housing were included, the aOR was 0.31 (0.19–0.49), *P*<0.001. Also, NH residents had a lower frequency of unplanned ER visits (model 4b), with an aOR of 0.44 (0.26–0.75), *P* = 0.002 (Table 4).

**Table 4:** Variables associated with unplanned ER visits during the last month of life among patients with ALS. Model 4a: Univariable and multivariable models for patients who live in their own homes (*n* = 349, of which 144 persons made at least one ER visit), NH residents were excluded (*n* = 99). Model 4b: Multivariable model for all patients, including NH residents (*n* = 448, of which 174 persons made at least one ER visit).

Variable	Model 4a Univariable analysis. NH residents excluded, (*n*=349)		Model 4a^e^ Multivariable analysis. NH residents excluded, (*n*=349)		Model 4b^e^ Multivariable analysis, NH residents included, (*n*=448)	
	OR^a^ (95% CI)	*P*	aOR^b^ (95% CI)	*P*	aOR^b^ (95% CI)	*P*
**Age groups (years)**						
18–64	Ref.		Ref.		Ref.	
65–74 ≥75	1.13 (0.68–1.88) 1.09 (0.62–1.91)	0.62 0.77	1.03 (0.60–1.77) 0.79 (0.43–1.45)	0.92 0.44	1.08 (0.65–1.79) 0.88 (0.50–1.53)	0.79 0.64
**Sex**						
Men	Ref.		Ref.		Ref.	
Women	1.31 (0.86–2.02)	0.21	1.38 (0.87–2.20)	0.17	1.06 (0.70–1.58)	0.79
**Socio-economic status**^c^ Mosaic group 1+2 Mosaic group 3	Ref. 1.04 (0.66–1.65)	0.87	Ref. 1.08 (0.65–1.80)	0.77	Ref. 0.86 (0.56–1.33)	0.50
**HFRS^d^** 1 (not frail) 2+3 (frail)	Ref. 2.41 (1.55–3.75)	**<0.001**	Ref. 2.46 (1.54–3.93)	**<0.001**	Ref. 2.22 (1.46–3.39)	**<0.001**
**Dementia** No Yes	Ref. 1.62 (0.64–4.11)	0.30	Ref. 0.98 (0.36–2.64)	0.97	Ref. 0.88 (0.40–1.93)	0.75
**SPC** No Yes	Ref. 0.33 (0.21–0.51)	**<0.001**	Ref. 0.31 (0.19–0.49)	**<0.001**	Ref. 0.38 (0.25–0.58)	**<0.001**
**Nursing home resident**						
No Yes	N/A in this model		N/A in this model		Ref. 0.44 (0.26–0.75)	**0.002**

a OR, odds ratio;

b aOR, adjusted odds ratio;

c socio-economic status: Mosaic groups 1+2 are more advantaged groups, Mosaic group 3 is a less advantaged group.

d HFRS, hospital frailty risk score.

e C statistic was 0.63 and 0.64 for models 4a and 4b, respectively.

Significant values (*P*<0.05) are written in bold.

### Acute hospitals as place of death

Persons with higher HFRS were more likely to die in acute hospitals, both as regards persons in ordinary housing (model 5a), as well as when NH residents were included (model 5b). In the total material, the aOR was 2.05 (1.27–3.32), *P* = 0.004. Those who received SPC were significantly less likely to die in acute hospitals, aOR 0.12 (0.07–0.20), *P*<0.001 (Table 5).

**Table 5:** Variables associated with acute hospitals as place of death among patients with ALS. Model 5a: Univariable and multivariable models for patients who live in their own homes (*n* = 349, of which 110 persons died in acute hospitals), NH residents were excluded (*n* = 99). Model 5b: Multivariable model for all patients, including NH residents (*n* = 448, of which 135 persons died in acute hospitals).

Variable	Model 5a Univariable analysis. NH residents excluded, (*n*=349)		Model 5a^e^ Multivariable analysis. NH residents excluded, (*n*=349)		Model 5b^e^ Multivariable analysis, NH residents included, (*n*=448)	
	OR (95% CI)	*P*	aOR^b^ (95% CI)	*P*	aOR^b^ (95% CI)	*P*
**Age groups (years)**						
18–64	Ref.		Ref.		Ref.	
65–74 ≥75	1.09 (0.64–1.87) 1.08 (0.60–1.96)	0.74 0.80	0.89 (0.48–1.65) 0.73 (0.36–1.44)	0.70 0.36	1.00 (0.56–1.80) 0.65 (0.35–1.24)	0.99 0.20
**Sex**						
Men	Ref.		Ref.		Ref.	
Women	0.88 (0.56–1.39)	0.58	0.92 (0.55–1.56)	0.76	0.82 (0.52–1.30)	0.41
**Socio-economic status^c^** Mosaic group 1+2 Mosaic group 3	Ref. 0.72 (0.43–1.19)	0.20	Ref. 0.65 (0.36–1.16)	0.14	Ref. 0.71 (0.43–1.18)	0.19
**HFRS^d^** 1 (not frail) 2+3 (frail)	Ref. 1.87 (1.17–2.97)	**0.009**	Ref. 2.06 (1.21–3.52)	**0.008**	Ref. 2.05 (1.27–3.32)	**0.004**
**Dementia** No Yes	Ref. 1.63 (0.64–4.16)	0.31	Ref. 0.74 (0.25–2.15)	0.57	Ref. 0.61 (0.28–1.58)	0.36
**SPC** No Yes	Ref. 0.15 (0.10–0.25)	**<0.001**	Ref. 0.11 (0.06–0.19)	**<0.001**	Ref. 0.12 (0.07–0.20)	**<0.001**
**Nursing home resident**						
No Yes	N/A in this model		N/A in this Model		Ref. 0.44 (0.24–0.78)	**0.005**

a OR, odds ratio;

b aOR, adjusted Odds ratio;

c socio-economic status: Mosaic groups 1+2 are more advantaged groups, Mosaic group 3 is a less advantaged group.

d HFRS, hospital frailty risk score.

e C statistic was 0.78 and 0.79 for models 5a and 5b, respectively.

Only 12% of those who received SPC died in acute hospitals, compared with 48% for others (Table 3). aOR for those in ordinary housing was 0.11 (0.06–0.19), *P*<0.001. As regards the whole material, also NH residents were less likely to die in acute hospitals, with an aOR of 0.44 (0.24–0.78), *P* = 0.005 (Table 5).

## Discussion

Our results show that the access to SPC in patients with ALS was equal in relation to gender, socio-economic standing, frailty, and age up to 75 years, whereas patients older than 75 years, and those with dementia and/ or living in NH residents were less likely to receive SPC. However, persons with ALS and a concomitant diagnosis of dementia are seldom candidates for SPC, as this kind of care is in most cases provided in the patient´s own home, and as a prerequisite for this type of care is that the patient can communicate and knows a certain degree of self-care.

Access to SPC reduced ER visits and deaths in acute hospitals. However, patients who were frail, had a higher risk of ER visits and were more likely to die in an acute hospital setting. NH residents were less likely to have ER visits and to die at hospital. Even though some of the results show equal care, our findings also indicate that the receipt of SPC in ALS is lower for some patient groups. It may be relevant with targeted SPC to meet the patients’ needs, as SPC obviously has advantages also for patients with ALS (14, 15).

Equal care, and thus, also receipt of SPC, is an important goal in the Swedish healthcare system and in the National Care Program for Palliative Care (26, 27). Our results indicate that equal care mostly is provided in practice. It may depend on the tax-funded healthcare system that offers public healthcare to all inhabitants, regardless of socioeconomic status or gender. This is in contrast with a study from USA, which shows that minorities, men, and unmarried people more often die in an acute care facility (20).

However, our results showed that those 75 years or older, as well as patients with dementia did not receive SPC to the same extent. This is of importance since both our own data on ALS and other palliative care research show that receipt of SPC is associated with a lower proportion of unplanned ER visits and death in acute hospital setting, both in cancer and non-cancer diagnoses (28) and receipt of palliative care is associated with better quality of care in ALS (14, 15). Furthermore, previous studies on other diagnoses have shown that symptom relief is superior and end of life discussions/advance care planning is more often provided in SPC settings, compared with hospital care for example, in dying persons of chronic obstructive pulmonary disease (COPD) (29), of cancer (30) or of COVID-19 and cancer (31). Therefore, all patients with ALS, regardless of age, should be offered SPC when needed, as a complement to the ALS-teams, especially in the end of life (14).

Frailty (HFRS) is a variable that is seldom discussed in ALS and to the best of our knowledge it has not been studied as regards healthcare utilization. However, being frail increased the likelihood to ER visits and death at an emergency hospital in our study, well in line with studies for example on cancer (32, 33). In our data, frailty was independently associated with healthcare utilization, also when controlled for example for age, sex or for being a nursing home resident. Thus, awareness of the importance of frailty in patients with ALS should increase. Moreover, frailty is an important differential diagnosis, as both frailty and ALS cause increasing weakness in the legs in elderly persons, and there is a risk that ALS is overlooked as a diagnosis. In fact, late age onset of ALS seems to be more common than formerly assumed, therefore, ALS needs to be seen as a potential differential diagnosis especially in older patients (34).

Patients with ALS, living at NH residents were older and the likelihood of ER visits and to die at the hospital was lower than for the others. This might have several explanations. Possibly, elderly persons with limited medical but with extensive nursing needs were more likely to be referred to NHs, which are mainly staffed by auxiliary nurses. Alternatively, both the medical and nursing care in NHs has a sufficiently high quality to avoid unplanned ER visits or hospitalizations. However, according to our clinical experience it is possible that their symptoms and other problems might be overlooked. Moreover, 20% of them eventually die in an acute hospital, implying that care at NHs would benefit from being supported with the expertise from an ALS or SPC team.

The evidence that patients with ALS who received SPC had less ER visits, fewer hospital admissions and more seldom hospital as place of death suggests that SPC probably provides a care that reduce the need of emergency care in the end of life. These results agree with a study in brain tumors (35), other cancer forms (28, 36), as well as with studies on chronic obstructive lung disease and heart failure (28, 37).

Previous studies have shown that symptom relief in the last week in life is inferior in patients with ALS than in cancer (6, 8). It has also been shown that partners of patients with ALS have a higher mortality due to external causes, including suicide and accidents, after the ALS diagnosis (38), and that both patients and relatives are emotionally and existentially affected by the disease (11–13). Thus, it might be beneficial with SPC from the patient’s perspective, to reduce suffering and increase symptom relief, but possibly also from a family perspective, as family support is an integral part of SPC (14, 15, 39). In addition, it is of importance that decision-makers understand the benefits of SPC, and thus prioritizes it, as the quality of palliative care is highly rated, both by patients and family members (21), in parallel with a reduced need of acute hospital admissions. However, in contrast to diagnoses such as cancer, a proportion of patients with ALS will die a sudden death (17), sometimes even before an admission to SPC is needed. For this reason, not all patients will be candidates for SPC.

## Strengths and limitations

The VAL database has few missing values, and the study is relatively large, as most studies on ALS and palliative care are qualitative studies (typically 10–20 informants) or quality-of-life studies (typically less than 100 patients) (12, 13), although occasional register studies exist (6, 8). It provides the opportunity to study the care utilization and shortcomings in the given care. However, a limitation is that we just know where the patients received their care, not in what extent it was preferred. Another limitation is that some of the presented associations are weak, despite statistical significance.
